# BWD-DETR: A Robust Framework for Bright-Field Wafer Defect Detection

**DOI:** 10.3390/s26031064

**Published:** 2026-02-06

**Authors:** Ruilou Zhang, Xiangji Guo, Yuankang Xu, Tianyu Zhang, Ming Ming

**Affiliations:** 1 Hangzhou Institute for Advanced Study, University of Chinese Academy of Sciences, Hangzhou 310024, China; zhangruilou23@mails.ucas.ac.cn (R.Z.); guoxiangji@ucas.ac.cn (X.G.); xuyuankang24@mails.ucas.ac.cn (Y.X.); zhangtianyu23@mails.ucas.ac.cn (T.Z.); 2 University of Chinese Academy of Sciences, Beijing 100049, China; 3 Shanghai Institute of Technical Physics, Chinese Academy of Sciences, Shanghai 200083, China

**Keywords:** wafer, defect detection, bright-field imaging

## Abstract

Optical defect detection based on bright-field imaging is currently one of the most widely applied inspection techniques in wafer fabrication. However, particle defects on the surface of patterned wafers are often small in size. Under bright-field optical imaging conditions, defect signals are easily overwhelmed by complex background textures and noise, seriously affecting the detectability and positioning accuracy of defects. To address this issue, this paper proposes BWD-DETR, a detection framework tailored for wafer surface defects under bright-field imaging. Based on the RT-DETR baseline, this framework integrates a wavelet backbone, an SMFI module, and a CAS-Fusion module, achieving an AP_50_ of 96.56% and an AP_50:95_ of 54.94% in bright-field wafer defect detection, with improvements of 1.64% and 2.17% over the baseline, respectively. The proposed method can effectively enhance the detection capability for sub-micron defects on the wafer surface.

## 1. Introduction

Due to the continuous scaling down of semiconductor process nodes and the rapid increase in circuit integration density, the tolerance for minute defects has significantly decreased. These defects not only lead to a decline in product yield but can also induce device performance degradation and reliability issues.

Currently, wafer defect detection in the semiconductor manufacturing field mainly relies on electron beam-based detection systems and optical imaging-based detection systems. Electron beam inspection is a typical detection method of scanning electron microscopy (SEM), and it is widely used in the detection of nanoscale defects due to its high resolution [1,2]. However, during electron beam inspection, its field of view is far smaller than that of optical inspection equipment. This leads to a detection efficiency that is significantly lower than optical far-field inspection, making it difficult to meet the requirements for rapid global wafer inspection. It is worth noting that SEM possesses a certain degree of destructiveness. Under conditions using relatively high beam currents and electron acceleration voltages, SEM analysis carries the potential risk of causing damage to the surface of the tested sample [3]. Optical microscopes can image large areas at high speeds due to their large imaging field of view [4]. In dark-field measurements, the presence of thermal effects may also cause changes in sample performance and even lead to its damage [3]. Bright-field detection, on the other hand, features a large imaging field of view and low exposure dose, enabling non-destructive and rapid detection.

Random defects are inevitably generated during the patterned wafer manufacturing process, and these defects are mainly caused by particle contamination adhering to the wafer surface. Due to the highly random nature of particle distribution, the specific locations of particle defects are difficult to predict in advance [5]. In large-scale, high-volume integrated circuit production, a significant portion of yield loss can be attributed to random particulate defects. Due to the small size of particulate defects, as well as the limitations of detection system resolution and the influence of noise, defect signals are often easily obscured by the intricate background textures of patterned wafers and system noise. Currently, the main detection methods are primarily based on image comparison approaches, including die-to-die [6], cell-to-cell [7], and die-to-database [8]. These methods identify and detect defects by performing differential calculations between defect areas and normal areas based on the difference results. In recent years, with the emergence of new technologies such as deep learning, new approaches have been introduced for defect detection in patterned wafers [9]. Deep learning can effectively enhance the accuracy and robustness of detection through automatic feature learning, enabling it to meet the demands of semiconductor manufacturing for large-scale, real-time, and automated detection.

However, many wafer defect detections based on deep learning target macroscopic defects, which are approximately hundreds of micrometers in size. These defects can be observed under a common low-power microscope, and their detection is relatively simple. For microscopic defects, most deep learning-based defect detection studies utilize SEM imaging to acquire data. Under bright-field imaging conditions, research on deep learning-based detection of micro-defects on patterned wafer surfaces remains relatively limited and is currently in its nascent stage [10]. Possible reasons include: there is currently a lack of publicly available datasets, and data may also be inaccessible due to confidentiality restrictions [4]; the annotation process for micro-defect datasets on patterned wafer surfaces under bright-field imaging is relatively complex, and collecting sufficient data for training is also time-consuming. Furthermore, when using simulated optical images as data, it is difficult to simulate system biases and random perturbations.

Unlike the high resolution of SEM, the resolution of bright-field optical microscopy is constrained by Rayleigh scattering and the diffraction limit [11]. Consequently, defects cannot be clearly imaged, and their signals are easily masked by noise. Particularly in the presence of complex background patterns, defect features are prone to being confused with background textures, thereby increasing the difficulty of precise defect detection. This paper proposes an improved object detection algorithm based on RT-DETR, named BWD-DETR (Bright-field Wafer Defect Detection Transformer), which aims to enhance the detection capability for defects with low signal-to-noise ratios. The main contributions of this paper are as follows: (a) We independently constructed a bright-field optical imaging system in a cleanroom environment to acquire real images of patterned wafer surfaces. Using the system, data samples were collected, annotated, and quality-checked, ultimately creating a high-quality dataset for wafer defect detection under bright-field imaging conditions, providing a reliable foundation for subsequent algorithm training and performance evaluation. (b) Introduced a wavelet-transform-based information retention mechanism to more effectively extract high-frequency, fine-grained defect features, improving the model’s sensitivity to tiny defects. (c) This paper proposes the SMFI module to enhance the model’s contextual modeling capability, thereby better capturing the dependencies between local and global defect regions. (d) A CAS-Fusion module is designed to enhance the discriminability of cross-scale feature fusion. This module adaptively emphasizes defect-related channels and strengthens local spatial representation capabilities through shift convolution.

The remainder of this paper is organized as follows: Section 2 introduces related work; Section 3 describes the proposed method in detail; Section 4 presents the experimental details and validation process, followed by an analysis of the results; Section 5 concludes the paper.

## 2. Related Work

### 2.1. Wafer Defect Detection Method Based on Deep Learning

In recent years, deep learning has gradually become an important development direction for wafer surface defect detection. Ameri et al. surveyed a large volume of relevant literature published in recent years, showing that deep learning has formed a rich methodological system in surface defect detection [12]. Yin et al. proposed a self-supervised contrastive learning framework integrating global and local representations for the classification and segmentation of mixed-type wafer defects. By introducing a local contrastive module, their method effectively addresses the loss of fine-grained details in pixel-level segmentation inherent in traditional contrastive learning, significantly improving performance under limited labeled data [13]. Cheon et al. proposed an automatic defect classification method based on CNN, which can achieve efficient wafer surface defect recognition without additional feature extraction and detect defects that do not occur in the training set [14]. Chen et al. proposed a die defect detection method combining GAN and YOLOv3. By generating diverse pseudo-defect images to improve YOLOv3 detection accuracy, it can achieve efficient detection without relying on a large number of real defect samples and without the need for additional annotation of pseudo-defects, making it suitable for various die patterns [15]. Choi et al. proposed a wafer defect detection method based on a Siamese network, achieving high-precision defect identification by comparing a golden die with the die under test, and applied Bayesian learning to improve the performance of the proposed Siamese network [16]. Wang et al. proposed MS-SANET, which integrates self-attention mechanisms and multi-scale feature extraction strategies into the U-Net architecture. Combined with the Log-Cosh Dice loss function, this method significantly enhances the capability to capture defects with weak textures and irregular shapes in semiconductor laser chips [17]. Xu et al. proposed the DYRT-DETR method, which effectively improves the detection performance for small particles and slender scratches on wafer surfaces through attention mechanisms, residual fusion feature pyramids, and dynamic snake convolutions [18]. Chen et al. proposed SR-FABNet, which effectively addresses the challenge of detecting nanoscale defects in low-resolution bright-field wafer images by innovatively combining a super-resolution auxiliary branch, fourier attention mechanism, and knowledge distillation [10].

### 2.2. RT-DETR Model

In recent years, the architecture based on the Transformer has demonstrated significant potential in industrial object detection tasks. Wang [19] demonstrated superior detection performance by integrating YOLOv10 with transformer-based backbone networks in the task of UAV-based rebar counting. Real-Time Detection Transformer (RT-DETR) is the first real-time end-to-end object detection framework [20]. Unlike traditional CNN-based detectors, RT-DETR uses Transformer [21] as its core architecture, performing end-to-end object prediction directly on the entire image, avoiding post-processing steps like Non-Maximum Suppression. RT-DETR consists of a backbone, an efficient hybrid encoder, and a Transformer decoder with auxiliary prediction heads. For the backbone, RT-DETR mainly uses CNN networks, such as ResNet [22]. The efficient hybrid encoder consists of Attention-based Intra-scale Feature Interaction (AIFI) and CNN-based Cross-scale Feature Fusion (CCFF). AIFI introduces a self-attention mechanism on high-level feature maps, achieving efficient intra-scale semantic modeling while enhancing object localization and recognition capabilities, while reducing computational overhead. CCFF uses convolutional fusion blocks to integrate adjacent scale features, improving cross-scale feature expression and small object detection performance while maintaining a lightweight design. RT-DETR improves inference speed through the efficient hybrid encoder, enhances detection accuracy using minimum uncertainty query selection, and supports a flexible balance between speed and accuracy by adjusting the number of decoder layers, thereby achieving real-time performance while maintaining the advantages of end-to-end detection.

## 3. Method

**Figure 1:**
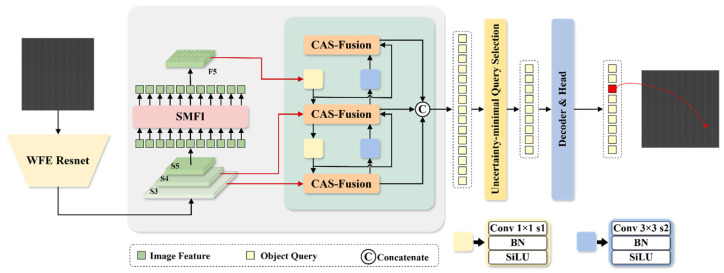
Overall framework of the proposed network.

This section presents our BWD-DETR framework and elaborates on three innovative modules, aiming to address the difficulty in detecting low signal-to-noise ratio defects on patterned wafer surfaces. BWD-DETR is an end-to-end object detector that utilizes an improved ResNet-18 as its backbone. Its backbone incorporates the wavelet transform as a frequency-domain feature extraction mechanism, which avoids the information loss inherent in traditional downsampling. By virtue of the adaptive sparse self-attention and local mixed-scale interaction mechanism of the SMFI module, the interference from irrelevant regions and noise is mitigated, and the capability of defect feature modeling is enhanced. The CAS-Fusion module is employed to achieve efficient multi-scale fusion, directing the model to focus more intensively on the channel responses of defects. Through frequency-domain information extraction, defect feature enhancement, and precise multi-scale fusion, the three modules form a synergistic closed loop with complementary functions. The overall framework of BWD-DETR is illustrated in Figure 1.

### 3.1. Backbone

**Figure 2:**
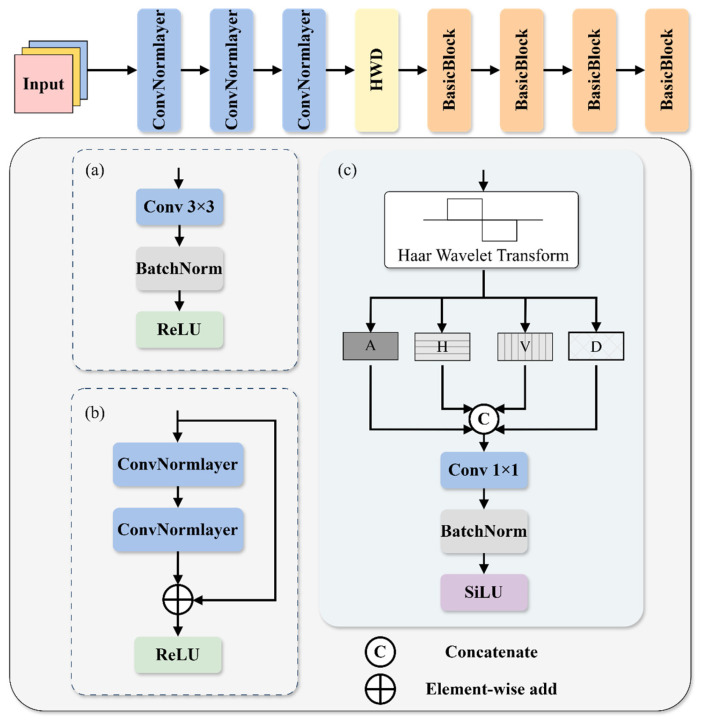
The structure of WFE ResNet. (**a**) The structure of ConvNormlayer. (**b**) The structure of Basicblock. (**c**) The structure of HWD.

For bright-field wafer defect detection, detailed information such as boundaries and textures is of vital importance in the accurate identification and location of defects. In addition, defects are mostly presented as subtle geometric disturbances and micro-scale texture changes, which essentially belong to high-frequency information. There are differences in information between defects and normal patterns. To this end, this paper proposes a wavelet-based feature extraction ResNet (WFE ResNet) backbone network. We introduced Haar Wavelet-based Downsampling (HWD) [23] into the original backbone network and replaced the final ReLU activation function with the SiLU activation function to enhance the expressive power of the model. The structure of WFE ResNet is shown in Figure 2. Among them, HWD can retain defect information as much as possible while reducing the spatial resolution of the feature map, and can enhance the extraction of high-frequency defect information, thereby improving the perception ability of the backbone network for wafer surface defects.

Haar wavelet is a fast decomposition method that provides multi-resolution representation and captures both the low-frequency and high-frequency components of an image simultaneously. The wavelet basis function and scaling function corresponding to the one-stage, one-dimensional Haar transform can be defined as follows:

(1) $$\left\{\begin{array}{cc} \phi_{1}\left(x\right)=\frac{1}{\sqrt{2}}\phi_{1,0}\left(x\right)+\frac{1}{\sqrt{2}}\phi_{1,1}\left(x\right) & \\ \psi_{1}\left(x\right)=\frac{1}{\sqrt{2}}\phi_{1,0}\left(x\right)-\frac{1}{\sqrt{2}}\phi_{1,1}\left(x\right) & \end{array}\right.$$

Here, $\phi_{j,k}(x)$ is defined as:

(2) $$\phi_{j,k}\left(x\right)=\sqrt{2^{j}}\phi \left(2^{j}x-k\right),k=0,1,\cdots ,2^{j}-1$$

The parameters j and k respectively represent the scale and direction of Haar basis functions in the field of image processing.

**Figure 3:**
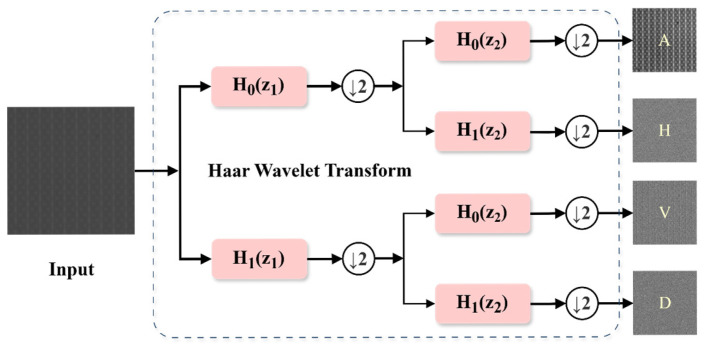
Schematic diagram of feature decomposition using haar wavelet transform.

The Haar wavelet downsampling module first uses the Haar wavelet transform to generate four components, among which one component is the low-frequency approximate component (A), and the other three are high-frequency detail components in the horizontal (H), vertical (V), and diagonal (D) directions. The spatial resolution of each component is half of the original image, while the number of channels of the feature map is four times that of the original. The Haar wavelet transform can encode part of the spatial dimension information into the channel dimension without losing any original information. Figure 3 shows the process of decomposing a wafer image with a resolution of *H* × *W* using the Haar wavelet transform. Among them, the symbol ↓2 indicates a downsampling operation, *H*_0_ indicates low-pass filtering of the original data, and *H*_1_ indicates high-pass filtering of the original data, which are respectively used to extract the approximate information and high-frequency information of the image. Subsequently, the four components obtained from the Haar wavelet decomposition are concatenated in the channel dimension to achieve fusion of multi-band features.

Since the Haar wavelet transform expands the number of channels of the input feature map by four times, if these four times the channels are directly passed into the subsequent network, it will lead to a sharp increase in the computational load and the number of parameters. At the same time, in order to adjust the number of channels of the feature map to align with the subsequent layers, a standard 1 × 1 convolutional layer is adopted for information fusion and channel compression. The features after convolution fusion are normalized to a unified distribution through BatchNorm(BN), thereby stabilizing the training process. Finally, the SiLU activation function is adopted to introduce nonlinearity for feature mapping. Meanwhile, by taking advantage of its characteristic of allowing negative values, the high-frequency texture details generated by the Haar wavelet transform are fully retained. The output feature F of the module can be expressed as:

(3) $$F={SiLU(BN(Conv}_{1\times 1}(Concat(A,H,V,D))))$$

### 3.2. SMFI Module

In the scenario of bright-field wafer defect detection, defects usually present low significance, weak structural disturbances and local texture anomalies, and the background area accounts for a large proportion. However, the multi-head self-attention operation of the AIFI module still performs intensive calculations on all spatial positions, which not only leads to computational redundancy but also makes it vulnerable to large-scale background noise interference. In addition, in the AIFI module, FFN mainly achieves semantic transformation through channel mapping and nonlinear activation. However, it does not explicitly utilize spatial neighborhood information, making it difficult for AIFI modules to fully explore the local context relationships of minor defects under complex wafer patterns.

**Figure 4:**
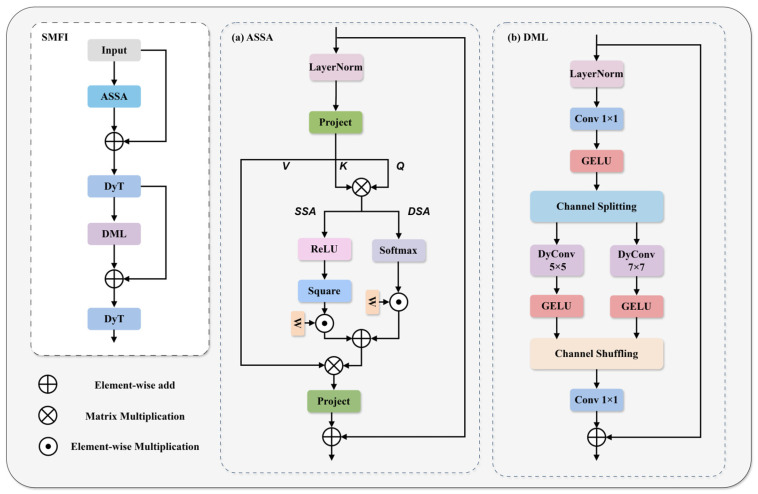
The structure of the SMFI module. (**a**) The structure of the ASSA module. (**b**) The structure of the DML module.

In response to these problems, this paper proposes the SMFI (sparse-enhanced mixed-scale feature interaction) module, whose structure is shown in Figure 4. This module enhances the feature focusing ability of the key defect area through Adaptive Sparse Self-Attention (ASSA) and suppresses redundant global modeling. To overcome the limitations brought by statistical calculation, we introduced Dynamic Tanh (DyT) [24]. This is a statistics-free element-by-element transformation that dynamically adjusts the input distribution using a learnable scaling factor α and uses the saturation characteristic of the Tanh function to perform nonlinear compression on extreme activation values. Finally, the Dynamic mixing layer (DML) is utilized to combine the mixed-scale dynamic convolution with the dynamic fusion weights. The efficient internal interaction of features is promoted through channel segmentation and rearrangement, improving the accuracy of local context modeling. The entire processing flow can be expressed as:

(4) $$F^{\prime }=DyT\left(F_{in}+ASSA\left(F_{in}\right)\right)$$

(5) $$F_{out}=DyT\left(F^{\prime }+DML\left(F^{\prime }\right)\right)$$

ASSA is one of the important components of SMFI. It adopts a dual-branch parallel architecture, aiming to balance the sparse selection of features with global perception. Specifically, this module consists of a sparse self-attention branch (SSA) for suppressing background and noise and a dense self-attention branch (DSA) for maintaining information flow.

In order to explicitly filter out the background regions and noise irrelevant to the defects, the sparse branch adopts the squared rectified linear unit operation. Unlike softmax, which assigns non-zero probabilities to all tokens, squared ReLU can force negative or low-correlation attention scores to zero. SSA can be expressed as:

(6) $$SSA=ReLU^{2}\left(\frac{{QK}^{T}}{\sqrt{d}}+B\right)$$

Here, we obtain the query (Q), key (K), and the subsequently used value (V) embeddings by linearly projecting the input features. B is the learnable relative position bias, and d is the dimension of the key vector.

Relying solely on sparse attention may lead to oversparsity in the feature map, which in turn causes potential problems such as loss of pattern structure information or gradient interruption. Therefore, ASSA parallelism introduces a dense branch based on a softmax layer. This branch is responsible for capturing global context dependencies, ensuring that while filtering out irrelevant regions and noise, the overall structure of the wafer image and long-distance semantic associations are retained. DSA can be expressed as:

(7) $$DSA=Softmax\left(\frac{{QK}^{T}}{\sqrt{d}}+B\right)$$

The final attention matrix can be expressed as:

(8) $$\begin{array}{c} A=\left(w_{1}*SSA+w_{2}*DSA\right)V \end{array}$$

(9) $$w_{n}=\frac{e^{a_{n}}}{\sum_{i=1}^{N}e^{a_{i}}},n=\left\{1,2\right\}$$

where $w_{1}$, $w_{2}$ ∈ $R^{1}$ are the two normalized weights of the adaptive modulation dual-branch, and {$a_{1}$, $a_{2}$} are learnable parameters. Through the initialization of one branch, this structure can balance noise suppression and information utilization, and flexibly adjust the sparsity of tokens [25].

DML is another important component of SMFI. DML achieves context modeling at mixed scales through channel splitting, dynamic convolution at different receptive field scales, and channel shuffling mechanisms. This design enables the network to capture the short-distance detail texture dependency and long-distance structural semantic dependency of bright-field wafer images in parallel at the same feature level. To enhance the nonlinear feature representation capability of deep networks, a GELU activation function is introduced in each branch after dynamic convolution [26]. The processed features are then concatenated and the channel sequence is reorganized using the channel shuffling operation to facilitate the information exchange between features of different scales. The built-in dynamic convolution mechanism of DML utilizes global average pooling (GAP) and linear projection to adaptively generate convolution kernel weights based on the semantic content of the input image, enabling the model to adopt different filtering strategies for the background and defect areas, thereby enhancing the model’s adaptability.

### 3.3. Fusion Module

In the specific scenarios of bright-field wafer defect detection, defects usually have the characteristics of being extremely small in scale and having very low contrast. The CCFF architecture of the original RT-DETR adopts the paradigm of Concatenation of channels followed by reconnection of parametric modules (RepBlock). This approach has limitations when dealing with fine-grained wafer defects. On the one hand, the concatenation operation only linearly stacks features in the channel dimension, lacking a context interaction mechanism between cross-scale features, making it difficult to fully exploit the potential of multi-scale architectures. On the other hand, although RepBlock is used for feature fusion to enhance expressive power, there is a certain degree of computational redundancy when dealing with low-contrast and complex-textured wafer backgrounds. Moreover, its standard convolution lacks an explicit mechanism to strengthen the spatial interaction between adjacent pixels when handling fine textures. It limits the model’s acute ability to capture high-frequency details such as defect edges.

**Figure 5:**
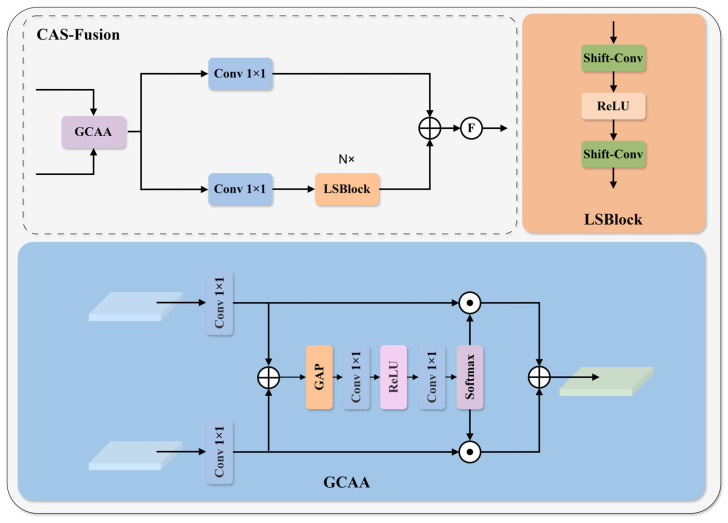
The structure of CAS-Fusion.

To break through this bottleneck, this paper proposes an efficient context-aware shift Fusion (CAS-Fusion) module, whose structure is shown in Figure 5. This module utilizes the designed global context-aware aggregation (GCAA) unit to achieve channel-level adaptive fusion of multi-scale features through a global prior. It also integrates a local shift block (LSBlock), which expands the effective receptive field at a low computational cost through parameter-free shift operations and accurately captures local structural information.

As the pre-core unit of CAS-Fusion, GCAA aims to address the semantic gap issue during the physical fusion of multi-scale features. Given the two heterogeneous input features $X_{1}\in R^{C_{1}\times H\times W}$ and $X_{2}\in R^{C_{2}\times H\times W}$ derived from different hierarchical levels for the GCAA module, we first employ branch-specific 1 × 1 convolutional projection functions $P_{i}(\cdot )$ to map these features into a unified semantic space C. This operation is designed to eliminate discrepancies in the input features along the channel dimension. Subsequently, the aligned features are superimposed to generate a mixed feature representation M that encapsulates multi-source information:

(10) $$M=\sum_{i=1}^{2}P_{i}\left(X_{i}\right)$$

Here, $M\in R^{C\times H\times W}$ aggregates spatial details and semantic contexts from distinct scales.

To capture long-range channel dependencies, Global Average Pooling (GAP) is first applied to the mixed feature M, compressing the two-dimensional spatial information into channel descriptors possessing a global receptive field. Subsequently, a multi-layer perceptron incorporating a bottleneck structure is utilized to model the non-linear interactions between channels. Finally, a softmax function generates the normalized adaptive weight vectors corresponding to the two branches:

(11) $$\left[\alpha_{1},\alpha_{2}\right]=Softmax\left(MLP\left(GAP\left(M\right)\right)\right)$$

where $\alpha_{i}$ denotes the importance weight for each channel in the i-th branch.

Finally, the generated weight vectors are employed to perform channel-wise dynamic recalibration on the original aligned features. The fused output feature Y is obtained via the weighted summation of the features from each branch:

(12) $$Y=\sum_{i=1}^{2}\alpha_{i}\cdot P_{i}\left(X_{i}\right)$$

Through this mechanism, the network can adaptively enhance the contribution of critical feature channels based on the semantic content of the input images, such as defect size and texture.

**Figure 6:**
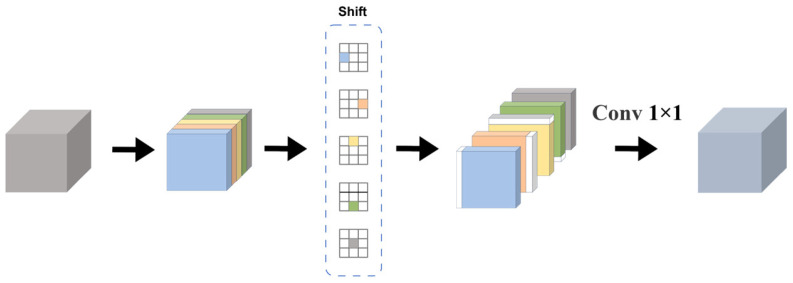
Schematic diagram of shift-conv.

Furthermore, CAS-Fusion incorporates the designed LSBlock module, wherein the core shift-conv [27] utilizes a parameter-free spatial shifting operation as an efficient alternative to traditional spatial convolutions. Figure 6 depicts a schematic diagram of shift-conv. Specifically, the shift-conv within the LSBlock partitions the feature maps into five groups along the channel dimension. The first four groups perform spatial shifts in the four cardinal directions (top, bottom, left, and right), while the fifth group retains the original spatial information. This mechanism achieves cross-pixel spatial information flow and aggregation with zero learnable parameter overhead. Subsequently, a 1 × 1 convolution is applied to facilitate channel-wise information mixing.

In terms of modular architecture, the LSBlock employs two cascaded shift-conv units to first project features into a high-dimensional space for non-linear transformation, and subsequently compress them back to the original dimension. This holistic design ensures that during the fusion process, features not only efficiently exchange channel information but also establish dense neighborhood dependencies within the spatial dimension. By fully interacting with local spatial details, it significantly enhances the network’s capability to capture defect edges and morphological nuances on the wafer surface, all while maintaining the lightweight nature of the module.

## 4. Experimental Results and Discussion

### 4.1. Bright-Field Imaging Detection Systems and Datasets

**Figure 7:**
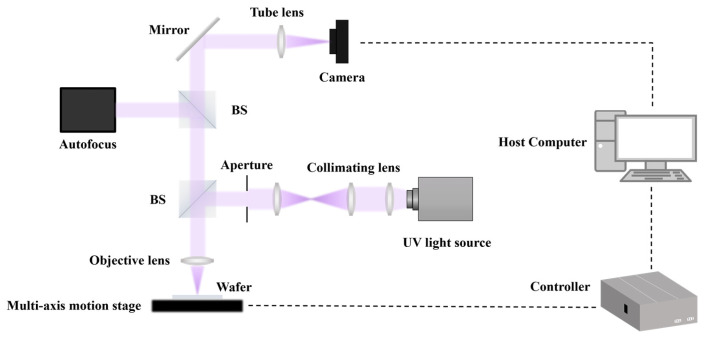
Wafer defect detection system based on bright-field imaging.

Due to the lack of publicly available datasets for wafer defect detection under bright-field imaging, this paper obtained high-quality real wafer surface images based on a self-built bright-field imaging detection system to construct the datasets required for the experiment. The system is mainly composed of an optical microscopic imaging unit, an illumination unit, an imaging acquisition module and a motion platform. Figure 7 presents the schematic of the wafer defect detection system based on bright-field imaging that we established. In the imaging section, a self-designed ultraviolet objective lens was selected as the front-end imaging element in the experiment to ensure the resolution capability for sub-micron defects. Its magnification was 200 times and the numerical aperture was 0.83. The illumination system adopts a stable UV mercury lamp and Kohler lighting solution, combined with optical path shaping to achieve uniform bright-field lighting, thereby ensuring the clarity and stability of wafer imaging. The imaging acquisition part is composed of high-resolution industrial area array cameras, with a sensor pixel size of 2.78 μm and a resolution of 2848 × 2848. After the camera is matched with the objective lens, it can achieve high-precision imaging of the wafer surface, ensuring the visibility of defects and the effectiveness of subsequent detection. Meanwhile, we use the ACS controller to drive the displacement platform to achieve high-precision horizontal movement, and combine it with the autofocus module to complete precise focusing in the Z-axis direction.

**Figure 8:**
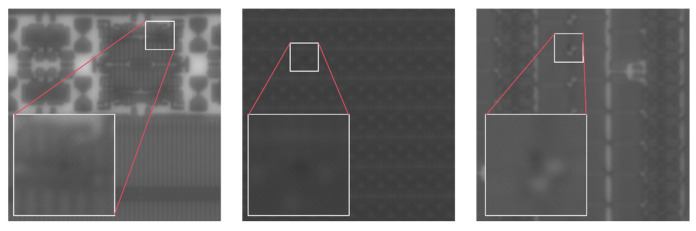
Sample images of the datasets.

We utilize the aforementioned system to acquire images. To mitigate the risk of overfitting, in addition to capturing images under standard wafer placement conditions, we also collect images under varying imaging conditions by applying slight rotations to the wafer and adjusting the exposure settings. The acquired images are then preprocessed with flat-field correction and other preprocessing steps. In this study, an image of size 2848 × 2848 was segmented into several 1024 × 1024 block images. To further enhance data diversity, we have also added a small number of images of other sizes. All the annotations were made after the original images were cropped. We used the LabelImg v1.8.6 software to label the defects in 1531 images. All image annotation work was conducted under the guidance of professionals and engineers from semiconductor companies to ensure the reliability of the dataset and the effectiveness of model training. The annotated original dataset was first divided into a training set, validation set and test set. We ensured that the images of wafer surfaces with different backgrounds in the training set, validation set, and test set are distributed as evenly as possible through artificial selection. Then, a small number of images in the original dataset were subjected to data augmentation processing, while strictly avoiding data leakage. The data augmentation methods included flipping, rotation, scaling, and adjusting brightness and contrast. We ultimately obtained a dataset containing 1851 images, with the ratio of the training set, validation set, and test set being 5:1:1, to achieve the goals of training, validation, and testing. Figure 8 presents the images of some samples.

### 4.2. Implementation Details and Metrics

The experimental platform of this article is equipped with AMD EPYC 9374F 32-Core Processor (Advanced Micro Devices, Inc., Santa Clara, CA, USA) and two NVIDIA GeForce RTX 5090 graphics cards with 32G RAM (NVIDIA Corporation, Santa Clara, CA, USA), and the operating system used is Ubuntu 22.04. This study is based on Pytorch as the deep learning framework, and the software used includes Python 3.11.13, PyTorch 2.7.1 and torchvision 0.22.1. During training, the constructed model utilized the AdamW optimizer with an initial learning rate of 0.0001. The network was trained with a total of 100 epochs, and the batch size was set to 4. The momentum factor was set to 0.9, and the weight decay was set to 0.0001. These parameter settings were determined to be the optimal values based on multiple experiments. We use the GIOU loss function [28] for bounding box regression.

To evaluate the performance of the model, this study adopted the average precision (AP), which is commonly used in object detection tasks, as the main measurement index, specifically including AP_50_ and AP_50:95_. AP_50_ represents the Average Precision calculated at an Intersection over Union (IoU) threshold of 0.5, corresponding to the area under the Precision-Recall (PR) curve. Meanwhile, AP_50:95_ denotes the mean of AP values calculated at IoU thresholds ranging from 0.50 to 0.95 with a step size of 0.05, which is utilized to evaluate the localization accuracy and robustness of the detection model in a more comprehensive and rigorous manner. The calculation formulas for precision (P), recall (R), and average precision (AP) are as follows:

(13) $$P=\frac{TP}{TP+FP}$$

(14) $$R=\frac{TP}{TP+FN}$$

(15) $$AP=\int_{0}^{1}P\left(R\right)dR$$

where TP denotes the number of samples correctly identified as defects, FP denotes the number of non-defective samples erroneously classified as defects, and FN denotes the number of actual defects missed by the model. Additionally, we introduce complexity metrics to further evaluate the performance of different models, including the number of parameters, computational cost, and inference time.

### 4.3. Performance Comparison of Different Object Detection Models

To verify the advancement of the BWD-DETR we proposed, a comparative experiment was conducted on the detection algorithms in recent years using the dataset we labeled. We selected a series of representative CNN and Transformer detectors for comparison, including YOLOv5m, YOLOv6n, YOLOv8m, YOLOv9m, YOLOv10m, YOLOv11m, Deformable DETR and DINO. As shown in Table 1, compared with the RT-DETR baseline model, BWD-DETR achieved performance improvements of 1.64% and 2.17%, respectively, on the AP_50_ and AP_50:95_ metrics, reaching 96.56% and 54.94%, while the inference time only increased slightly (1.2 ms). Moreover, the model we proposed outperforms YOLOv5m by 1.19% in the key metric AP_50:95_.

**Figure 9:**
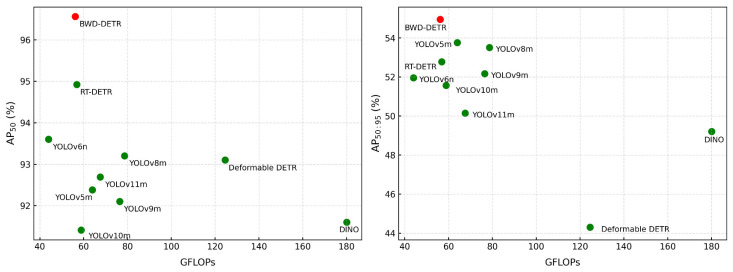
Comparison of complexity and performance of different models.

Figure 9 shows the comparison of computational complexity with AP_50_ and AP_50:95_. It can be seen that BWD-DETR also has an advantage in computational complexity. Its computational complexity is only higher than that of YOLOv6n, but it is 2.96% and 2.99% higher than YOLOv6n in the AP_50_ and AP_50:95_ metrics, respectively. The computational load of BWD-DETR is reduced by 0.7 GFlops compared to the baseline model. Although our model does not have a significant advantage over other comparison models in terms of inference time, it can still meet the actual deployment requirements of industrial scenarios. Most importantly, BWD-DETR performs the best among all comparison methods and is suitable for online detection of wafer defects.

### 4.4. Comparison of Different Backbone Networks

To verify the effectiveness of the proposed WFE ResNet, we compare it with several representative backbone networks widely adopted in recent detection frameworks. Table 2 presents the quantitative comparison of different backbone networks on the wafer defect detection task. It can be observed that while the RT-DETR models based on RepViT, ConvNeXtV2, and UniRepLKNet backbones successfully reduced computational cost and parameter counts, their detection performance suffered varying degrees of degradation. Although EfficientViT slightly outperforms our method in the AP_50_ metric, indicating its high defect detection capability, WFE ResNet achieves a significant 0.9% lead in the stricter AP_50:95_ metric. This result strongly demonstrates the advantage of the wavelet transform downsampling module in preserving high-frequency details of the image, thereby endowing the model with stronger, precise localization ability. Additionally, MobileNetV4′s AP_50:95_ also exceeds the baseline ResNet18, but WFE ResNet still maintains the highest AP_50:95_ across the board. Compared with the original backbone, WFE ResNet consistently improves detection accuracy under the condition of consistent parameter quantity and almost no loss in inference speed. This indicates that enhancing feature representation by introducing wavelet domain information is a more effective defect detection solution than simply stacking convolutional layers or using larger models such as SwinTransformer.

### 4.5. Comparison of Different AIFI Improvement Methods

To verify the effectiveness of the improved AIFI we proposed for wafer defect detection under bright-field imaging, we respectively utilized DAttention, EAA, and HiLo to replace the multi-head attention mechanism in AIFI. The input features are first processed by these modules to extract attention-aware representations; the resulting features are then added to the original input and followed by the first layer normalization. Subsequently, the normalized features are fed into a feed-forward network composed of two convolutions, and the output is obtained after another residual connection and a final layer normalization. The results in Table 3 show that the SMFI module we designed achieved the highest AP_50_ (0.9583) and AP_50:95_ (0.5353) without significantly increasing the detection time, the number of parameters, and the computational load. This indicates that the proposed SMFI module can effectively enhance the feature interaction capability and noise robustness, highlighting its advantages in patterned wafer surface defect detection under bright-field imaging.

### 4.6. Visualization and Analysis

**Figure 10:**
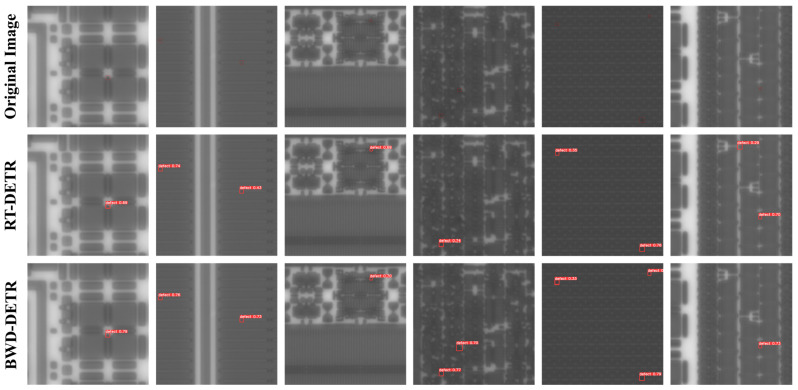
Visualization of RT-DETR and BWD-DETR detection results.

Figure 10 shows partial detection results of the proposed model, including wafer surface images with diverse background patterns and structural complexities. For clarity, ground-truth defects in the original images are marked with red bounding boxes. The second and third rows present the detection results of RT-DETR and BWD-DETR, respectively. The experimental results indicate that the improved model consistently yields higher confidence scores for defect predictions compared to the baseline. This is primarily attributed to the enhanced feature extraction and representation capabilities introduced by the proposed modules, which enable the model to more effectively distinguish defects from complex backgrounds and thereby reduce uncertainty caused by ambiguous regions.

Observing the results in the fourth and fifth columns, it can be seen that the baseline model suffers from missed detections, indicating its limited capability in detecting small defects under complex background patterns. In contrast, the proposed model successfully detects these defects that the baseline fails to identify. Furthermore, as shown in the last column, the baseline model misclassifies normal pattern structures as defects, whereas our improved model correctly distinguishes genuine defects from regular patterns. Despite the challenges posed by the extremely small defect size, low contrast against intricate backgrounds, and susceptibility to various types of noise, the proposed framework consistently achieves stable and effective defect detection on patterned wafers under bright-field imaging conditions. Visualization-based detection results show that the improved model still achieves higher confidence scores on these challenging samples, indicating its enhanced capability to capture high-frequency features and its more robust ability to suppress background clutter and noise, which effectively increases the detection recall and reduces false positives.

**Figure 11:**
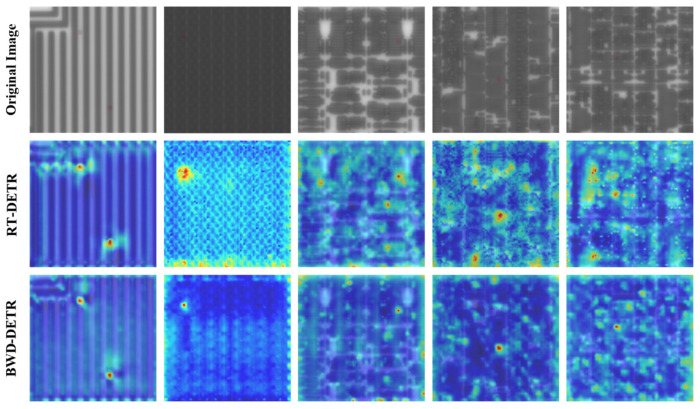
Visualization results of the heatmap.

To further evaluate the detection performance of the BWD-DETR algorithm under bright-field imaging conditions, we utilized the Grad-CAM++ [29] heatmap technique to visualize the feature activation regions of the baseline model and the improved model. The results are shown in Figure 11, where the first row represents the original images, the second row shows the heatmaps generated by RT-DETR, and the third row presents the heatmaps generated by our proposed model. The red parts in the figure indicate the regions in the original images that trigger stronger network responses and make greater contributions. Through this intuitive method, the target sensitivity before and after improvement can be compared. From the results of the first and second columns, it can be seen that both the baseline and improved models can have good activation responses to defect regions in images with simple patterns and large structural sizes. However, the activation range of the improved model is compact and the heat intensity is strong, while the large-area background textures are effectively suppressed. In the third to fifth columns, the wafer patterns are more complex. In the heatmaps of the original RT-DETR model, it can be observed that some background regions show high responses, and the thermal distribution at the boundaries of defect targets is rather blurry. This indicates that the model still has deficiencies in feature discrimination, which may increase the risk of false detections. In contrast, our model significantly reduces false activations in background regions, maintains close attention to defect areas, effectively highlights defects and suppresses background noise, and the distribution of heat concentration regions aligns more closely with the actual target locations.

### 4.7. Ablation Experiment

We conducted ablation experiments on the proposed model to verify the performance advantages brought by different improved methods. The results of the ablation experiments are shown in Table 4. The experiment systematically evaluated the individual contributions and synergistic effects of the three core improved methods by adding individual modules and combining multiple ablation modules.

As shown in Table 4, using the WFE ResNet backbone improves AP_50_ by 0.78% and AP_50:95_ by 0.6%. These performance improvements are attributed to HWD’s ability to extract and perceive high-frequency defect features, as well as its ability to faithfully preserve all extracted information. After using the SMFI module, the model improved by 0.91% and 0.76%, respectively, in the AP_50_ and AP_50:95_ metrics, indicating that the SMFI module can enhance global dependency and interaction, significantly improving the feature expression ability. Adding the CAS-Fusion module alone increases AP_50_ and AP_50:95_ by 1.01% and 1.31%, respectively. Subsequently, we conducted experiments using both the WFE ResNet and the SMFI module simultaneously. Compared to using either component alone, their combination yields greater performance gains: AP_50_ and AP_50:95_ improve by 0.94% and 1.59%, respectively, over the baseline model. This demonstrates a significant synergistic effect between the two modules. Specifically, WFE ResNet enables lossless feature downsampling via wavelet transform, preserving both high- and low-frequency structural information; building upon this, the SMFI module performs adaptive attention-based interaction and dynamic fusion, thereby achieving collaborative enhancement of texture details and high-level semantic features. The complete BWD-DETR framework achieves an AP_50_ of 96.56% and an AP_50:95_ of 54.94%, representing a significant improvement over the baseline model. This demonstrates the critical role of the CAS-Fusion module, which performs adaptive fusion and structural reconstruction of multi-scale features to provide the detection head with unified, high-quality feature representations.

In addition, we conducted ablation experiments on the CAS-Fusion module, and the results are shown in Table 5. Adding the GCAA module alone increased AP_50_ and AP_50:95_ by 0.56% and 0.48%, respectively; after adding the LSBlock, AP_50_ and AP_50:95_ rose by 0.83% and 1.1%, respectively. The significant improvement in AP_50:95_ indicates that shift-conv can effectively expand the receptive field and enhance the defect localization capability.

### 4.8. Generalization Experiments

The primary objective of model generalization experiments is to evaluate the adaptability of algorithms across diverse scenarios. Recently, Han et al. have conducted a series of studies focusing on industrial surface defect detection, proposing representative defect detection algorithms, including SA-FPN [30] based on the feature pyramid and the real-time detection model LSRF-YOLO [31], both of which have been validated on the public NEU-DET dataset [32]. This work also adopts the NEU-DET steel surface defect dataset to evaluate generalization performance. This dataset comprises 1800 images, covering six categories of defects: rolled-in_scale, inclusion, crazing, scratches, patches, and pitted_surface, with 300 images per category.

We randomly split the dataset into training, validation, and test sets in a 7:1:2 ratio. The detection results of the proposed method on this dataset are presented in Table 6. The experimental results show that, compared to the baseline model, BWD-DETR achieves improvements of 2.88% in mAP_50_ and 1.22% in mAP_50:95_, demonstrating that the proposed method effectively enhances detection performance across various datasets.

## 5. Conclusions

In this study, we independently developed a bright-field imaging-based wafer defect inspection system. Leveraging this experimental platform, we captured real images of wafer surfaces and constructed a high-quality wafer defect dataset accordingly. Furthermore, we propose BWD-DETR to address the specific challenges of detecting wafer surface defects under bright-field imaging. By integrating a Wavelet-based backbone with the SMFI and CAS-Fusion modules, our method effectively preserves high-frequency details while suppressing background interference. Experiments conducted on our dataset demonstrate that BWD-DETR achieves an AP_50_ of 96.56% and an AP_50:95_ of 54.94%, significantly outperforming the RT-DETR baseline. Visual heatmap analysis further validates the framework’s high robustness and capability for precise localization of tiny defects, even amidst complex background conditions.

In summary, our experiments have validated the effectiveness of the proposed method for the real-time defect detection of patterned wafers under bright-field imaging. Consequently, our deep learning-based solution for bright-field wafer defect detection demonstrates considerable potential for propelling advancements in automated optical inspection, semiconductor process control, and the forthcoming era of intelligent manufacturing. By delving into these realms, we aim to unlock new frontiers in real-time quality assurance and contribute significantly to the yield enhancement of critical semiconductor devices.

In future work, we intend to further optimize our bright-field imaging system to enhance imaging quality and enrich our dataset. We will also leverage CUDA acceleration and deploy the model with NVIDIA TensorRT on edge devices to achieve high-throughput, low-latency inference. Future work will delve into TensorRT quantization optimization and edge-side performance validation. Additionally, it should be noted that our current evaluation is limited to controlled bright-field imaging conditions, without a systematic assessment of the model’s sensitivity to common real-world perturbations such as sensor noise, illumination fluctuations, and contrast degradation. To better characterize its robustness and operational limits in practical semiconductor inspection scenarios, we plan to investigate BWD-DETR’s performance under controlled variations in lighting, exposure, sensor noise levels, and other imaging parameters. Simultaneously, we will focus on continuously refining the model architecture to address the increasingly complex challenges of wafer defect detection in bright-field imaging.

## Figures and Tables

**Table 1:** Comparative experimental results of different models.

Model	Params	GFlops	AP_50_	AP_50:95_	Inf Time
YOLOv5m	25.0 M	64.0	0.9238	0.5375	5.9 ms
YOLOv6n	16.3 M	44.0	0.9360	0.5195	4.1 ms
YOLOv8m	25.8 M	78.7	0.9320	0.5350	6.5 ms
YOLOv9m	20.0 M	76.5	0.9210	0.5216	7.6 ms
YOLOv10m	15.3 M	58.9	0.9141	0.5156	5.9 ms
YOLO11m	20.0 M	67.6	0.9269	0.5014	6.7 ms
DINO	47.5 M	180.1	0.9160	0.4920	29.9 ms
Deformable DETR	40.1 M	124.6	0.9310	0.4430	19.1 ms
RT-DETR	19.9 M	56.9	0.9492	0.5277	6.0 ms
Ours	20.5 M	56.2	0.9656	0.5494	7.2 ms

**Table 2:** Comparison of experiment results of backbone networks.

Method	Params	GFlops	AP_50_	AP_50:95_	Inf Time
ResNet18	19.9 M	56.9	0.9492	0.5277	6.0 ms
RepViT	13.3 M	36.3	0.8910	0.4852	6.5 ms
SwinTransformer	36.3 M	97.0	0.9273	0.4958	20.2 ms
EfficientViT	10.7 M	27.2	0.9585	0.5247	6.4 ms
MobileNetV4	11.3 M	39.5	0.9487	0.5330	4.2 ms
ConvNeXtV2	12.3 M	31.9	0.9457	0.5170	7.5 ms
UniRepLKNet	12.7 M	33.4	0.9220	0.5061	7.0 ms
WFE ResNet	19.9 M	57.8	0.9570	0.5337	6.4 ms

**Table 3:** Comparison of experiment results of different AIFI improvement methods.

Method	Params	GFlops	AP_50_	AP_50:95_	Inf Time
AIFI	19.9 M	56.9	0.9492	0.5277	6.0 ms
DAttention	19.9 M	57.2	0.9297	0.5169	5.7 ms
EAA	19.9 M	57.1	0.9265	0.4942	5.7 ms
HiLo	19.8 M	57.1	0.9531	0.5216	5.5 ms
SMFI	20.8 M	57.6	0.9583	0.5353	6.2 ms

**Table 4:** Results of ablation experiments.

Baseline	WFE ResNet	SMFI	CAS-Fusion	Params	GFlops	AP_50_	AP_50:95_	Inf Time
√				19.9 M	56.9	0.9492	0.5277	6.0 ms
√	√			19.9 M	57.8	0.9570	0.5337	6.4 ms
√		√		20.8 M	57.6	0.9583	0.5353	6.2 ms
√			√	19.6 M	54.6	0.9593	0.5408	6.7 ms
√	√	√		20.8 M	58.4	0.9586	0.5436	6.7 ms
√	√	√	√	20.5 M	56.2	0.9656	0.5494	7.2 ms

**Table 5:** The ablation experiment results of the CAS-Fusion module.

Baseline	GCAA	LSBlock	Params	GFlops	AP_50_	AP_50:95_	Inf Time
√			19.9 M	56.9	0.9492	0.5277	6.0 ms
√	√		19.7 M	55.6	0.9548	0.5325	6.1 ms
√		√	19.8 M	56.0	0.9575	0.5387	6.2 ms
√	√	√	19.6 M	54.6	0.9593	0.5408	6.7 ms

**Table 6:** Comparative experimental results of the proposed method on the NEU-DET dataset.

Model	Params	GFlops	mAP_50_	mAP_50:95_	Inf Time
RT-DETR	19.9 M	56.9	0.7258	0.4195	2.1 ms
BWD-DETR	20.5 M	56.2	0.7546	0.4317	3.4 ms

## Data Availability

The original contributions presented in this study are included in the article. Further inquiries can be directed to the corresponding author.
